# Juvenile pleomorphic adenoma of the cheek: a case report and review of literature

**DOI:** 10.1186/1746-1596-4-32

**Published:** 2009-09-22

**Authors:** Taufik Dalati, Mahmoud R Hussein

**Affiliations:** 1 Department of Oral & Maxillofacial surgery, Faculty of Dentistry, Damascus University Hospitals, Damascus, Syria; 2 Department of Pathology, Faculty of Medicine, Assuit University Hospitals, Assuit, Egypt

## Abstract

Pleomorphic adenoma, also called benign mixed tumor, is the most common tumor of the salivary glands. About 90% of these tumors occur in the parotid gland and 10% in the minor salivary glands. The most common sites of pleomorphic adenoma of the minor salivary glands are the palates followed by lips and cheeks. Other rare sites include the throat, floor of the mouth, tongue, tonsil, pharynx, retromolar area and nasal cavity. In children, intraoral pleomorphic adenomas of the cheek are extremely rare with only three cases reported to date. Here we report a case of pleomorphic adenoma of minor salivary glands of the cheek in a 17-year-old girl. The mass was removed by wide local excision with adequate margins, and after a follow-up period of three years there were no recurrences. To conclude, pleomorphic adenoma should be considered in the differential diagnosis of cheek masses in youngsters. Wide local excision is to be recommended as the treatment of choice. A close follow-up is necessary postoperatively.

## Introduction

Pleomorphic adenomas are benign salivary gland tumors that represent about 3- 10% of the neoplasm of the head and neck region [1]. They are the most common tumors (50%) of the major and minor salivary glands [2]. The palate is considered as the most common intraoral site (42.8-68.8%), followed by the upper lip (10.1%) and cheek (5.5%) [3-5]. Other rare sites include the throat (2.5%), retromolar region (0.7%), floor of the mouth and the alveolar mucosa [4]. Pleomorphic adenoma usually presents as a mobile slowly growing, painless firm swelling that does not cause ulceration of the overlying mucosa [6].

Pleomorphic adenoma consists of cells with epithelial and mesenchymal differentiation (mixed tumor). The highly variable morphology of this neoplasm is the result of interplay between these elements. Now it is widely accepted that both epithelial and mesenchymal (myxoid, hyaline, chondroid, osseous) elements often arise from same cell clone, which may be a myoepithelial or ductal reserve cell. There is no difference in behavior of this neoplasm based on proportion of various elements [7]. Lee et al examined formalin-fixed, paraffin-embedded tissues from 13 pleomorphic adenomas of female patients. They used the polymerase chain reaction (HUMRA assay). HUMARA, the human androgen receptor gene, is located on the X chromosome and contains a segment of polymorphic CAG tandem repeats in exon 1. Several methylation-sensitive HhaI restriction sites are located 5' to these CAG repeats. It is an ideal tool to study clonality of female tissues by examining the methylation pattern. A monoclonal pattern was seen in the stromal, epithelial elements in the majority of cases. These findings suggest that the stromal and epithelial cells in pleomorphic adenomas of salivary gland arise from the same clone in most cases [7]. Variants of pleomorphic adenoma include pleomorphic adenoma with lipomatous change [8], myxoliopmatous pleomorphic adenoma, pleomorphic adenoma with squamous differentiation and benign metastasizing mixed tumor [9].

The mucosa of the cheek is a uncommon site of occurrence for intraoral pleomorphic adenoma [10] and most of these cases have been reported in adults [11,12]. In children, only three cases were reported to date [13-15]. Here we report a case of pleomorphic adenoma in 17 years old girl. The relevant studies were discussed.

## Case report

**Figure 1:**
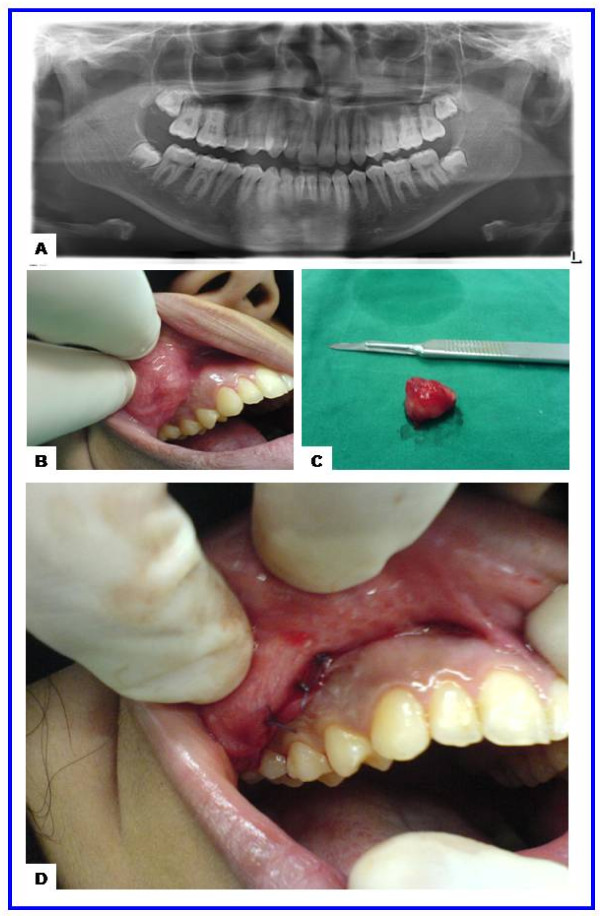
**The radiological and gross features of juvenile pleomorphic adenoma of the cheek**. **(a) **Panoramic radiograph showing unremarkable maxilla, mandible and teeth. **(b) **Preoperative view showing ovoid nodule in the right cheek covered by intact mucosa with congested blood vessels. **(c) **Gross features with a well circumscribed ovoid mass measuring 2.0 × 1.7 × 1.5 cm. **(d) **Postoperative view showing the site of the wound and sutures.

**Figure 2:**
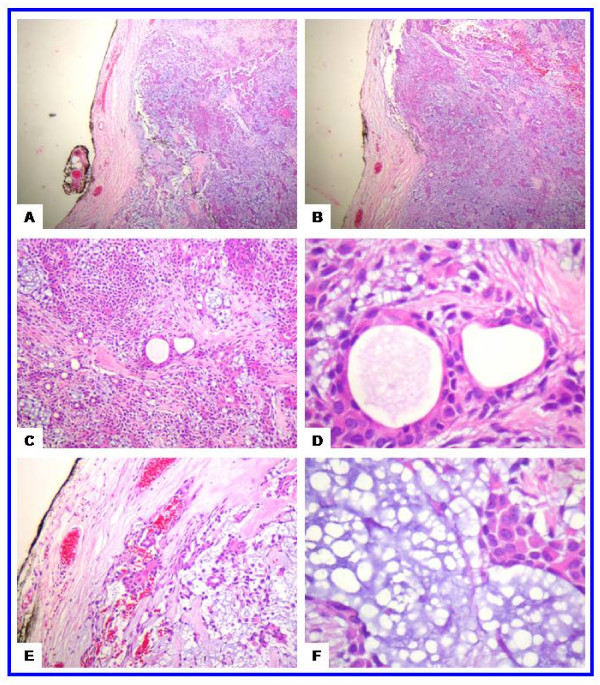
**The histological features of the juvenile pleomorphic adenoma of the cheek**. **(a-b) **Scanning magnification views of pleomorphic adenoma showing that the tumor is well circumscribed. The mesenchymal and epithelial elements are evident at these magnifications. This encapsulated tumor has a protuberance pushing through the capsule (upper left side). The tumor has abundant chondroid areas. **(c-d) **These views show the essential cytoarchitectural features sufficient for diagnosis of pleomoprhic adenoma. The tubular strucutes are well delineated from the stroma. They are lined by inner layer of ductal epithelial cells and other layer of myoepithelial cells. The latter merge imperceptivity into the surrounding mesenchymal elements. The tubules have eosinophilic colloid like materials (secretions) in their lumens. The myoepithelial cells appear as cuboidal, spindle, plasmacytoid and non-descript epithelioid cells. They surround the tubules in a thin layer and in focally thick mantles. **(e-f) **These views show the stroma (one of the defining component of the pleomorphic adenoma). It is composed of hyaline, homogenous, fibrillary (e) and chondromyoxoid (f) materials interspersed among the epithelial and myoepithelial cells.

A 17-year-old girl presented with a slowly growing painless swelling in the right cheek of four years duration. Clinical examination revealed a 2.0-cm, firm, mobile mass in the right cheek (2.0-cm dorsal to the angle of the mouth). There was no history of trauma, fevers, disturbance of salivation, or oral surgeries. The laboratory tests were unremarkable. Radiological examination showed no abnormality in the panoramic radiograph (Figure 1-A). The mass was dissected and excised with safety margins under local anesthesia. It did not involve the facial muscles or subcutaneous tissue of the cheek. Grossly, the lesion was in the form of an ovoid well demarcated, partially encapsulated, gray-white partly myxoid, partly rubbery mass, measuring 2.0 × 1.7 × 1.5 cm, with solid cut surface (Figure 1-B-F). On histology, a well-circumscribed growth was seen. The neoplastic proliferation had biphasic populations of epithelial and mesenchymal cells. The former was composed of glandular structures lined by round, oval cells having large hyperchromatic nuclei, pink cytoplasm and myoepithelial basal cell layer. The stroma was myxoid, hyaline and chondroid. No mitotic figures or necrosis were seen (Figure 2). Postoperative period was uneventful. The patient was followed up over a period of for 3 years and no recurrences were observed.

## Discussion

Pleomorphic adenoma occurs more frequently in women than in men and is most common from the fourth to sixth decades with a mean age of 43-46 years [3,4,16]. Salivary gland tumors are rare in children and when they do arise, they preferentially affect major salivary glands, but minor salivary gland tumors have also been reported [17]. Here we report a rare case of juvenile pleomorphic adenoma of the cheek in 17 years old girl. To the best of our knowledge, this is the fourth case to be reported in literature [13-15].

The clinicopathologic features of the case reported here concurs with previous studies. Yamamoto et el reported a 9-year-old Japanese girl with pleomorphic adenoma of the cheek mucosa. Cohen and Kronenberg reported two more cases of juvenile pleomorphic adenomas of the cheek (girls, age <18 years) [13-15]. No recurrence was reported in these cases [13-15]. Dhanuthai et al reported the first case of palatal pleomorphic adenoma in a 13 year-old child [17]. In children, Jorge et al reported five cases of intraoral pleomorphic adenoma in patients under 18 years of age in two Brazilian institutions. Four patients were females and one was male; two cases affected the palate, two the upper lip and one the tongue. The cases were treated by local excision and long-follow up showed no recurrences were observed. Jorge et al concluded that intraoral pleomorphic adenoma seems to have similar biological characteristics as in adults, with low recurrence rates after surgical resection [16]. The surgical treatment for the pleomorphic adenoma in both juvenile and adult patients is principally the same and includes complete wide surgical excision with good safety margins. Inadequate resection or rupture of the capsule or tumors spillage during excision can lead to local recurrence as these tumors often have microscopic interruptions in the capsule [16]. In adults, Van Heerden and his colleagues examined the clinicopathologic features of the oral salivary gland neoplasms. Seventy cases were diagnosed during 8-year period. Pleomorphic adenoma was the most common entity that accounted for 48% of all tumors (not in the cheek), but none of these lesions affected the cheek mucosa. Polymorphous low-grade adenocarcinoma comprised 15.7% of the tumors [3].

The differential diagnosis of the juvenile pleomorphic adenoma of the cheek (Table 1) includes buccal space abscess, dermoid cyst, foreign body reaction, fibroma, lipoma, neurofibroma, rhabdomyosarcoma, mucoepidermoid carcinoma, adenoid cystic carcinoma, polymorphous low-grade adenocarcinoma and carcinoma ex pleomorphic adenoma [16,18]. The possibility of buccal space abscess was ruled out due to absence of sign of inflammation. The solid nature of the lesion coupled with the lack of tissue representing the three germ layers rule out the possibility of mature cystic teratoma (dermoid cyst). The lack of ulceration of the buccal mucosa, pain, paresthesia or invasion of the surrounding tissue rules out the possibility of malignant transformation. Carcinoma ex pleomorphic adenoma is characterized by the presence of malignant epithelium (salivary duct carcinoma, undifferentiated carcinoma, adenocarcinoma not otherwise specified, terminal duct carcinoma or myoepithelial carcinoma) with benign stroma. Carcinoma ex pleomorphic adenoma is extensively infiltrative malignancy with necrosis, perineurial invasion, frequent mitotic figures, marked nuclear atypia. Adenoid cystic carcinoma usually shows cribriform, solid or tubular pattern similar to cylindromas of skin. It is composed of small bland myoepithelial cells with scant cytoplasm and dark compact angular nuclei that surround pseudoglandular spaces with PAS+ excess basement membrane material and mucin. Peripheral perineurial invasion and small true glandular lumina are sometimes seen but no squamous differentiation; or extensive necrosis are usually absent. Adenoid cystic carcinoma has high proliferative index, high p53 immunoreactivity, intense staining for BCL-2 but negative reactivity for glial fibrillary acidic protein. In contrast, pleomorphic adenoma is not invasive, show no perineurial invasion; has squamous metaplasia and mesenchyme-like areas. Charactaristically, pleomorphic adenoma has strong glial fibrillary acidic protein in the myxochondromatous areas [19]. The basal cell carcinoma is a low grade malignancy similar to basal cell adenoma. It is an infiltrative tumor with perineurial invasion and vascular invasion; variable cytologic atypia and mitotic activity. It is composed of solid, trabecular, tubular or membranous patterns but there is no myxoid matrix or cartilegenous areas. The basal cell adenoma is composed of basaloid cells sharply delineated from the stroma by basement membrane. The polymorphous low-grade adenocarcinoma is usually nonencapsulated tumor with diverse (polymorphous) growth patterns, infiltrative borders, perineurial invasion and rare tumor necrosis [3,20].

**Table 1:** Differential diagnosis of juvenile pleomorphic adenoma of the cheek

**Tumor**	**Salient histological features**
**Carcinoma ex pleomorphic adenoma**	

**Adenoid cystic carcinoma**	- Cribriform and pseudoglandular patterns of basaloid cells with hyalinized stroma - Frequent perineural invasion - Minimal nuclear pleomorphism - No squamous differentiation - No high grade dysplasia or squamous carcinoma in situ, no extensive necrosis

**Polymorphous low grade adenocarcinoma**	- Uniform plump columnar cells with bland nuclei arranged in variable growth patterns (tubular, cribriform, papillary, solid, fascicular, microcystic, single file, pseudoadenoid cystic, strand-like, mixed) - Perineural invasion common around small nerves - Infiltrative borders

**Basal cell adenoma**	- Elongated and branching canaliculi separated by hyaline stroma

**Acinic cell carcinoma**	- Variable cell types (serous, clear, vacuolated, intercalated duct cells) arranged in solid, microcystic, papillary cystic and follicular patterns - few mitotic figures

**Clear cell carcinoma-hyalinizing type**	-Trabeculae, cords, islands or nests of monomorphic clear cells surrounded by hyalinized bands with foci of myxohyaline stroma

**Cystadenocarcinoma**	- Invasive, cystic growth pattern, 75% had - Conspicuous papillary component - Composed of small cuboidal cells, large cuboidal cells, tall columnar cells or mixture - Cystic spaces with hemorrhage and granulation tissue are frequent

**Intraductal carcinoma**	- intraductal neoplasm with micropapillary, cribriform, solid, comedo or clinging patterns, with preservation of myoepithelial cells surrounding intraductal tumor
**Mucoepidermoid carcinoma**	- Cords, sheets, clusters of mucous, squamous, intermediate and clear cells; low to high grade
**Epithelial myoepithelial carcinoma**	- Both epithelial and myoepithelial components seen - Most tumor cells have myoepithelial features with clear cytoplasm or naked nuclei - Focally, there are ducts or tubules with an outer rim of myoepithelial cells and inner, dark ductal cells with scant eosinophilic cytoplasm and round, bland nuclei

## Conclusion

To conclude, juvenile pleomorphic adenoma of the cheek is a rare neoplasm and therefore its diagnosis requires a high index of suspicion. Complete wide surgical excision is the treatment of choice. Recurrence after many years of surgical excision as well as malignant transformation should be a concern and therefore long- term follow- up is necessary.

## Consent

Written informed consent was obtained from the patient for publication of this case report and accompanying images. A copy of the written consent is available for review by the Editor-in-Chief of this journal.

## Competing interests

The authors declare that they have no competing interests.

## Authors' contributions

TD outlined the general concept, was involved in the literature search, preparing the materials, supplying the relevant clinical details, providing the radiological details and clinical figures, drafting and revising the manuscript. MRH was involved in histopathology evaluation, interpreted the histopathology, providing the histopathology details, drafting and revising the manuscript. Both authors have read and approved the present manuscript.

## References

[B1] Garcia Berrocal JR, Ramirez Camacho R, Trinidad A, Salas C (2000). Mixed tumor (pleomorphic adenoma) of head and neck. Typical and atypical patterns. An Otorrinolaringol Ibero Am.

[B2] Traiger J, Rosen MB (1965). Mixed Tumor of the Cheek; Report of a Case. Oral Surg Oral Med OralPathol.

[B3] van Heerden WF, Raubenheimer EJ (1991). Intraoral salivary gland neoplasms: a retrospective study of seventy cases in an African population. Oral Surg Oral Med Oral Pathol.

[B4] Wang D, Li Y, He H, Liu L, Wu L, He Z (2007). Intraoral minor salivary gland tumors in a Chinese population: a retrospective study on 737 cases. Oral Surg Oral Med Oral Pathol Oral Radiol Endod.

[B5] Toida M, Shimokawa K, Makita H, Kato K, Kobayashi A, Kusunoki Y, Hatakeyama D, Fujitsuka H, Yamashita T, Shibata T (2005). Intraoral minor salivary gland tumors: a clinicopathological study of 82 cases. Int J Oral Maxillofac Surg.

[B6] Kaminski M, Janicki K (2002). A case of giant pleomorphic adenoma of the cheek with two malignant centers. Otolaryngol Pol.

[B7] Lee PS, Sabbath-Solitare M, Redondo TC, Ongcapin EH (2000). Molecular evidence that the stromal and epithelial cells in pleomorphic adenomas of salivary gland arise from the same origin: clonal analysis using human androgen receptor gene (HUMARA) assay. Hum Pathol.

[B8] Kondo T (2009). A case of lipomatous pleomorphic adenoma in the parotid gland. Diagn Pathol.

[B9] Ide F, Kusama K (2004). Myxolipomatous pleomorphic adenoma: an unusual oral presentation. J Oral Pathol Med.

[B10] Bablani D, Bansal S, Shetty SJ, Desai R, Kulkarni SR, Prasad P, Karjodkar FR (2009). Pleomorphic adenoma of the cheek: a case report and review. J Oral Maxillofac Surg.

[B11] Vyas KC, Mathur SP (1984). Pleomorphic salivary adenoma of cheek. A case report J Laryngol Otol.

[B12] Houston GD (2003). Oral pathology. Pleomorphic adenoma. J Okla Dent Assoc.

[B13] Yamamoto H, Fukumoto M, Yamaguchi F, Sakata K, Oikawa T (1986). Pleomorphic adenoma of the buccal gland in a child. Int J Oral Maxillofac Surg.

[B14] Cohen MA (1986). Pleomorphic adenoma of the cheek. Int J Oral Maxillofac Surg.

[B15] Kronenberg J, Horowitz A, Creter D (1988). Pleomorphic adenoma arising in accessory salivary tissue with constriction of Stensen's duct. J Laryngol Otol.

[B16] Jorge J, Pires FR, Alves FA, Perez DE, Kowalski LP, Lopes MA, Almeila OP (2002). Juvenile intraoral pleomorphic adenoma: report of five cases and review of the literature. Int J Oral Maxillofac Surg.

[B17] Dhanuthai K, Sappayatosok K, Kongin K (2009). Pleomorphic adenoma of the palate in a child: a case report. Med Oral Patol Oral Cir Bucal.

[B18] Baldus SE, Streppel M, Stennert E, Dienes HP (1999). Pleomorphic adenoma (mixed tumor) of the external auditory canal. Differential diagnosis of the tumors of the ceruminal glands. Pathologe.

[B19] Cerulli G, Renzi G, Perugini M, Becelli R (2004). Differential diagnosis between adenoid cystic carcinoma and pleomorphic adenoma of the minor salivary glands of palate. J Craniofac Surg.

[B20] Harada H (2000). Histomorphological investigation regarding to malignant transformation of pleomorphic adenoma (so-called malignant mixed tumor) of the salivary gland origin: special reference to carcinosarcoma. Kurume Med J.

